# ﻿Discovery and redescription of the true *Nuvolumbrosus* Navás and naming of a new *Nuvol* species (Neuroptera, Chrysopidae, Leucochrysini)

**DOI:** 10.3897/zookeys.1158.98572

**Published:** 2023-04-21

**Authors:** Francisco José Sosa-Duque, Catherine A. Tauber

**Affiliations:** 1 Universidade Federal Rural da Amazônia (UFRA) Campus de Capitão Poço, Pará, Brazil Universidade Federal Rural da Amazônia UFRA Campus de Capitão Poço Brazil; 2 Department of Entomology, Comstock Hall, Cornell University, Ithaca, NY 14853, USA Cornell University Ithaca United States of America; 3 Department of Entomology and Nematology, University of California, Davis, CA 95616, USA University of California Davis United States of America

**Keywords:** Chrysopinae, lacewing, misidentification, new species, taxonomy

## Abstract

Examination of a newly discovered specimen of *Nuvol* showed that our earlier species determination of *Nuvolumbrosus* Navás had been incorrect and that our “redescription” of the species actually applied to an undescribed species. Here, we redescribe the true *N.umbrosus*, based on a newly discovered male specimen. This specimen closely resembles Navás’ description, and it was collected from the Atlantic Forest as was the original type specimen. In addition, we assign the previously misidentified *Nuvol* specimens from the Amazonian region to a separate species, *Nuvolsatur* Sosa & Tauber, **sp. nov.** As a result of these actions, the genus *Nuvol* now contains two morphologically and geographically distinct species. In addition, the abdomens and genitalia of both sexes of *Nuvol* are now described (although each from a separate species).

## ﻿Introduction

The Neotropical green lacewing tribe Leucochrysini, a diverse and largely unstudied group in the neuropteran family Chrysopidae, currently contains ~190 described species uncomfortably classified into seven genera (Mantoanelli et al. 2006; Tauber 2007; Tauber et al. 2008; Breitkreuz et al. 2021). One of the small genera in the tribe is the monotypic *Nuvol*Navás (1916) – a genus that has remained largely unstudied because specimens are very few. Navás retained the type specimen of the type species, *Nuvolumbrosus* Navás, in his personal collection; it is missing (Monserrat 1985: 240; Tauber and Sosa 2015: 143). Also missing are two other specimens from Brasil: one from the state of Rio de Janeiro (Navás 1929a: 860, as “*Newol umbrosus*”; Navás 1929b: 319) and another from the state of São Paulo [photographed and studied by P. A. Adams at the Museu de Universidade de Zoologia da São Paulo (MZUSP), as reported by Brooks and Barnard (1990: 251, reference to P. A. Adams’ unpublished notes)]; see Tauber and Sosa (2015: 142)].

Approximately one hundred years after the species description, we discovered two female specimens from the Amazonian region that we tentatively identified as *N.umbrosus* Navás (Tauber and Sosa 2015). Based on our comparison of these two specimens with other leucochrysines, we concluded that aspects of the wing venation and a unique pattern of suffused banding on the wings were sufficient to warrant, at least temporarily, the retention of *Nuvol* as a valid genus within Leucochrysini (Tauber and Sosa 2015). However, we were not satisfied with our tentative determination of the two specimens as phenotypic variants of *N.umbrosus*. They exhibited several morphological features not reported for *N.umbrosus*, and they had been collected from sites far from the type locality. Thus, we continued to question if the two specimens actually represented a second species of *Nuvol*.

Recently, we discovered an additional specimen of *Nuvol* – a male in the collection at the Instituto de Ciências Biológicas, Universidade Federal de Minas Gerais, Belo Horizonte, Brazil. This specimen clearly fits Navás’ original description and drawing closely – much more closely than the specimens we had studied earlier. Its discovery indicated that Navás’ description and drawing were quite accurate, and that our hesitancy to firmly identify the Amazonian specimens as *N.umbrosus* was well founded. The specimen also indicated that our description and images of the Amazonian specimens depict a new, unnamed species in the genus.

Here, based on the newly found specimen, we first redescribe the true *N.umbrosus* Navás and provide information on the terminalia of a *Nuvol* male. Second, we correct the misidentification of our earlier specimens from the Amazonian region and recognize them as representing a new species. Finally, with the addition of male abdominal characteristics, we update the available diagnostic information for the genus *Nuvol* and briefly discuss the relationship of *Nuvol* with other leucochrysine genera.

## ﻿Materials and methods

The procedures used here were identical to those used in our previously published work, specifically: Tauber and Sosa (2015); Tauber et al. (2017).

Our abbreviations for museums are as follows:

**EMUS**Entomological Museum, Utah State University, Logan, Utah, USA;

**INPA**Coleção de Invertebrados do Instituto Nacional de Pesquisas da Amazônia,

Manaus, Amazonas, Brazil;

**MZUSP**Museu de Zoologia da Universidade de São Paulo, São Paulo, Brazil.

## ﻿Taxonomy

### ﻿ Nuvol umbrosus

Taxon classificationAnimaliaNeuropteraChrysopidae

Navás, 1916

D9DD5D02-C5E6-527A-8DD9-B534E6AFD8D0

Nuvol umbrosus Brotéria (Zoológica) 14: 25; “Rio de Janeiro, Febrero de 1912” (only one specimen). Navás 1929a: 860 (locality record, as Newol [sic] umbrosus); Navás 1929b: 319 (locality record); Penny 1977: 28 (species list); Brooks and Barnard 1990: 251 (taxonomy, drawing of wings from Adams’ notes on MZUSP specimen); Oswald 2013 (catalog listing); Tauber and Sosa 2015: 141–153 (taxonomic treatment based on incorrect species identification).

#### Redescription.

One male specimen preserved in alcohol, examined by FS: “MG, São Gonzalo Rio Abaixo, EA [Estação Ambiental, 19°53'2.86"S, 43°22'26.14"W, 751m] Peti, 30.iv.2012, A. F. Kumagai” (deposited in the collection of the Instituto de Ciências Biológicas, Universidade Federal de Minas Gerais (ICB – UFMG), Belo Horizonte, Brazil).

**Figure 1.**
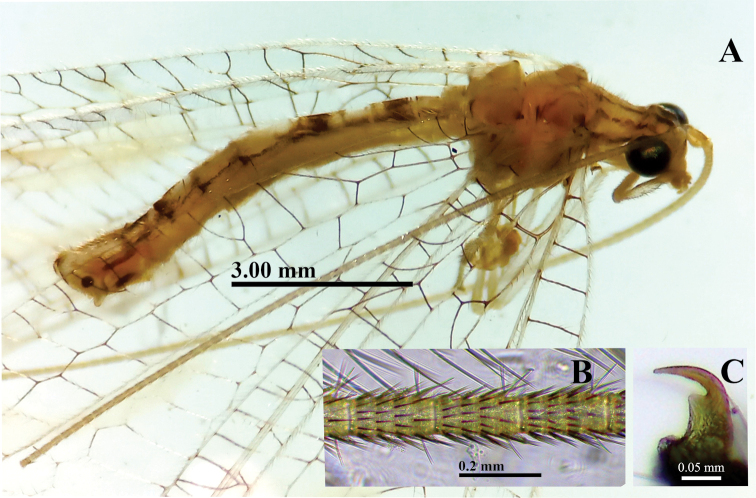
*Nuvolumbrosus* (Brazil, Minas Gerais) **A** habitus, dorsolateral **B** antennomeres ~ 1/3 distance from base of antenna **C** protarsal claw.

**Figure 2.**
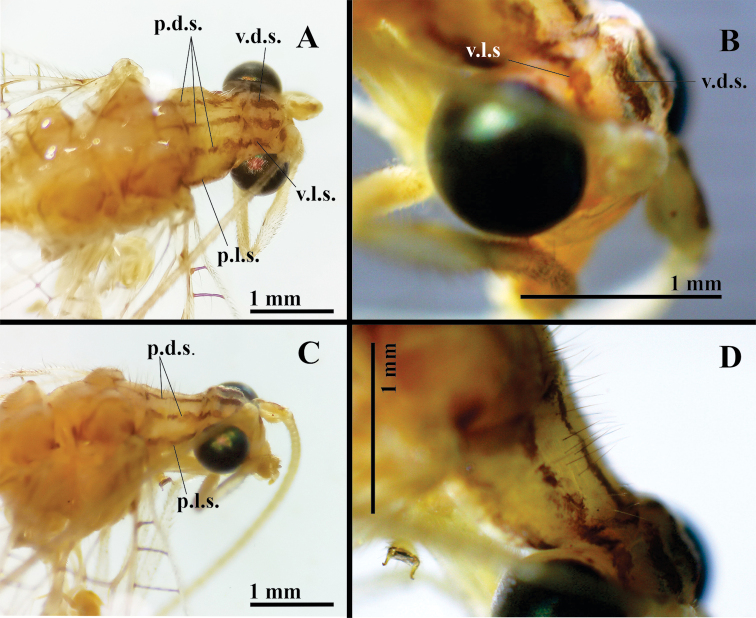
*Nuvolumbrosus* (Brazil, Minas Gerais) **A** head and thorax, dorsal **B** vertex, dorsolateral **C, D** head and thorax, dorsolateral. Abbreviations: **p.d.s.** pronotal dorsal stripe; **p.l.s.** pronotal lateral stripe; **v.d.s.** dorsal stripe on vertex; **v.l.s.** lateral stripe on vertex.

Note: Navás’ figure illustrated only the dorsal marks on the pronotum, not the lateral stripes; however, he explicitly mentioned the lateral stripes in his description. Thus, the specimen we describe here matches Navás’ type specimen in having five distinct dark red longitudinal stripes on the pronotum and two thin, somewhat diffuse, lateral stripes on the mesonotum and metanotum.

***Body*** (Fig. 1): Slender, yellowish to greenish, with elongate, slender antennae (both broken), hyaline wings marked with conspicuous brown to golden bands. ***Head*** (Figs 1A, 2A–C): Vertex raised, yellowish green, with two wide, reddish-brown longitudinal stripes dorsally, two smaller stripes laterally near edge of eyes. Frons, clypeus greenish; genae red. Labial, maxillary palpi yellow, unmarked. Antennae with scapes elongate, relatively large, close to each other mesally, cream colored, with light reddish-brown stripe dorsally (Fig. 2A, B); pedicel apparently unmarked; flagellar segments (basal section of flagellum) elongate, each with four swirls of robust, acute black setae (Fig. 1B). Measurements: head width (dorsal) 1.7 mm, ratio of head width / eye width 1:2.2; scape length 0.46 mm, width 0.35 mm.

***Thorax*** (Fig. 2A, C, D): Prothorax slightly wider than long (length 0.9 mm, width 1.2 mm), notum with five thin, longitudinal, reddish-brown stripes, three dorsal [as illustrated by Navás (1916)], two on lateral margin [absent from Navás’ drawing]; surface with elongate, golden setae mesolaterally. Mesothorax, metathorax with dark red marks laterally. Legs pale, without markings, with numerous light-brown to amber setae; tarsal claws with broad, dilated base, deep narrow cleft (Fig. 1C).

**Figure 3.**
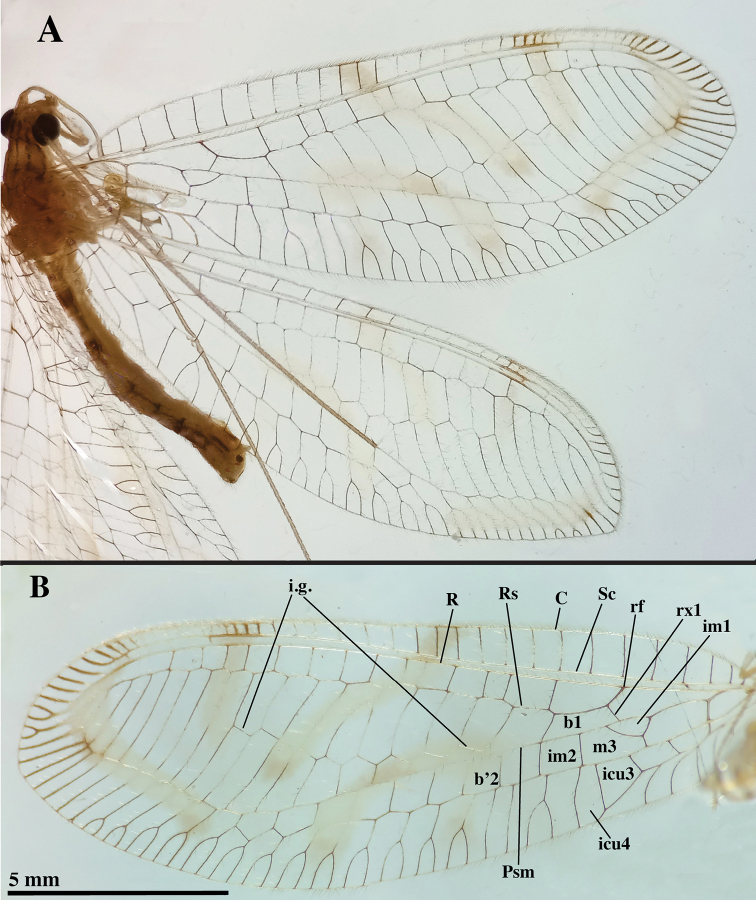
*Nuvolumbrosus* wings (Brazil, Minas Gerais) **A** right forewing and hindwing **B** left forewing with cells and veins identified. Note presence of apical veinlets with and without forks, markings, radius turning downward at tip of wing, forewing with four intracubital cells. Abbreviations: **b1** first upper Banksian cell; **b’2** second lower Banksian cell; **C** costa; **icu3, icu4** third and fourth intracubital cells; **im1, im2** first and second intramedian cells; **i.g.** inner gradate series; **m3** third median cell; **Psm** pseudomedia; **R** radius; **Rs** radial sector; **rf** origin of radial sector; **rx1** first radial crossvein; **Sc** subcosta.

***Wings*** (Fig. 3): Forewing 17.0 mm long, 6.1 mm wide (at widest point), with ratio of length / maximum width = 2.8:1; width at midpoint 5.6 mm, width along distal margin of basal quadrant, 4.2 mm; at base of distal quadrant 5.7 mm. Costal area relatively narrow; tallest costal cell (#8) 0.9 mm tall, with height 1.5 times its width, 0.16 times width of wing (midwing). First intramedian cell (*im1*) triangular, height at base (along median arculus, ma) 0.46 mm, width 2.1 times height, 0.57 times width of third median cell (*m3*). First radial crossvein distal to origin of radial sector (Rs); radial area (between R and Rs) with single row of 14 short, closed cells; tallest radial cell (*ra-rp1*) 0.69 mm in height, 0.72 times shorter than its width; two *b* cells (cells beneath Rs, not including an inner gradate vein); eight *b*’ cells (cells beneath Psm, after *im2*). Nine discrete inner gradates in regularly ascending, almost linear pattern, basal one not reaching Psm. Nine to eleven outer gradates aligned in relatively straight line adjacent to margin of wing, from tip of Psm to tip of Rs. Height of fourth inner gradate cell 1.1 times width. Four intracubital cells (*icu1-icu3* closed, *icu4* open). Subcosta, radial sector forked apically; thirteen to fourteen posterior terminal veins forked, distal six simple, without forks. Longitudinal veins, crossveins simple, slender, largely without crassate sections. Alar membrane with three large, conspicuous, diffuse, light yellowish-brown marks; stigma brown marked (Fig. 3B). Most veins dark, those beneath diffused alar markings appearing hyaline.

Hindwing 15.8 mm long, 5.1 mm wide. Nine discrete inner gradates, basal one not reaching Psm. Six outer gradates ascending in relatively straight to slightly zigzag trajectory adjacent to wing margin. Thirteen radial cells (counted from origin of radius, not false origin). Two large *b* cells (no small “*t*” cell); seven *b*’ cells beyond *im2*. Membrane with yellowish-brown diffused marks, similar to those on forewing; veins generally dark, but light in areas of diffused markings; stigma with single, weak brown spot basally, brown veins.

**Figure 4.**
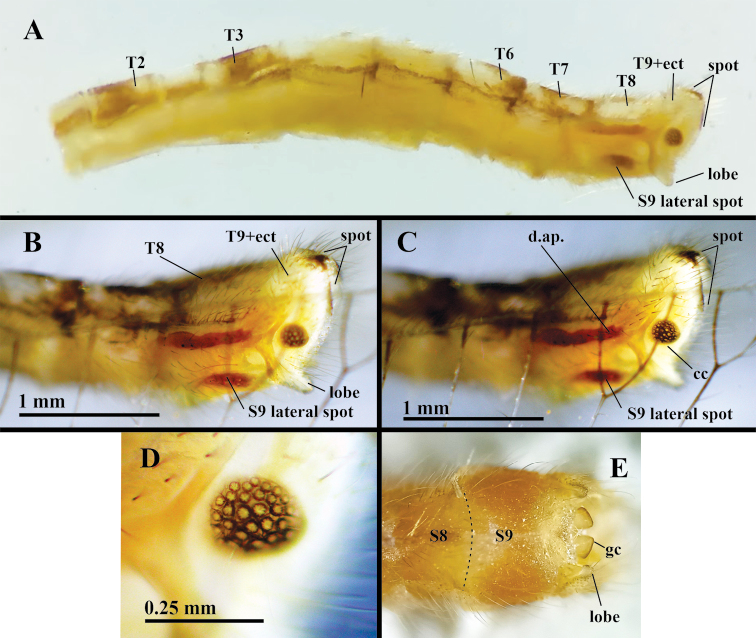
*Nuvolumbrosus* male abdomen (Brazil, Minas Gerais) **A–C** uncleared abdomen, lateral. Note apical lobe on distoventral corner of T9+ect with dense field of long, robust setae. Setae surrounding dorsal apodeme below T8, lateral spot on S9 **D** callus cerci and trichobothria (setae obscured) **E** S8+9, ventral. Note exposed gonocornua at apex of S9; dashed line showing possible suture scar between S8 and S9. Abbreviations: **cc** callus cerci; **d.ap.** dorsal apodeme; **gc** gonocornu; **S8, S9** eighth and ninth sternites; **T2–T8** second to eighth abdominal tergites; **T9+ect** fused ninth tergite and ectoproct.

**Figure 5.**
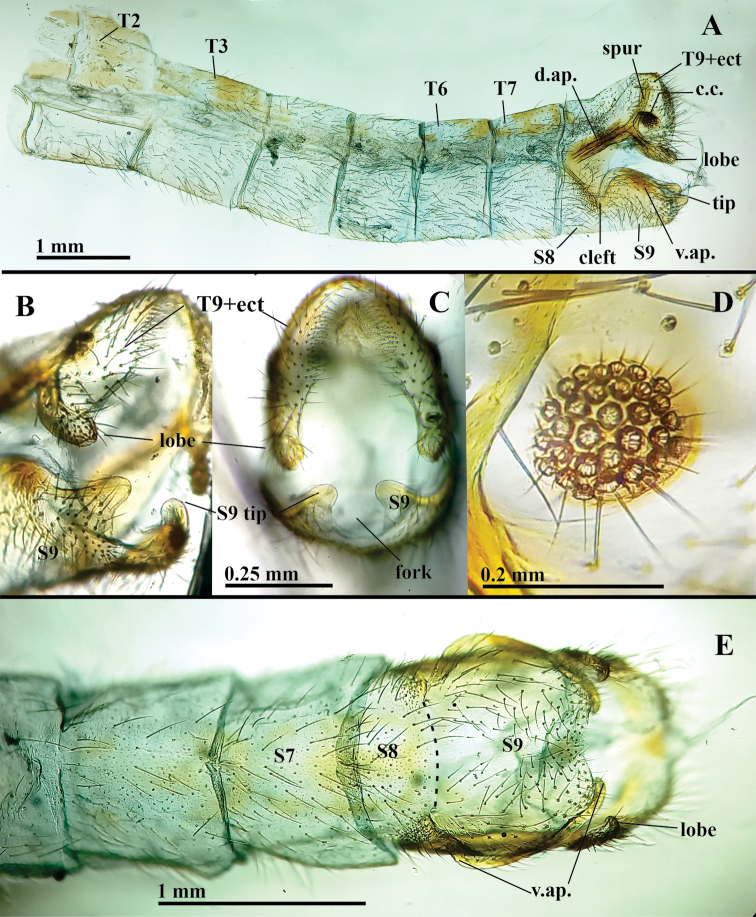
*Nuvolumbrosus* male terminalia cleared, with genitalia removed (Brazil, Minas Gerais) **A** abdomen, lateral **B** abdominal tip, posterolateral **C** abdominal tip, caudal **D** callus cerci **E** terminal segments, ventral [**Note for (E)**: S6–S8 densely covered by microtrichiae; dashed line between S8 and S9 indicating a possible suture scar; S9 bearing long, robust setae and lacking microtrichiae.] Abbreviations: **c.c.** callus cerci; **d.ap.** dorsal apodeme; **lobe** setose lobe at distal apex of dorsal apodeme; **S7, S8, S9** seventh, eighth and ninth abdominal sternites; **T2, T3, T6, T7** second, third, sixth, seventh abdominal tergites; **T9+ect** fused ninth abdominal tergite and ectoproct; **v.ap.** ventral apodeme.

**Figure 6.**
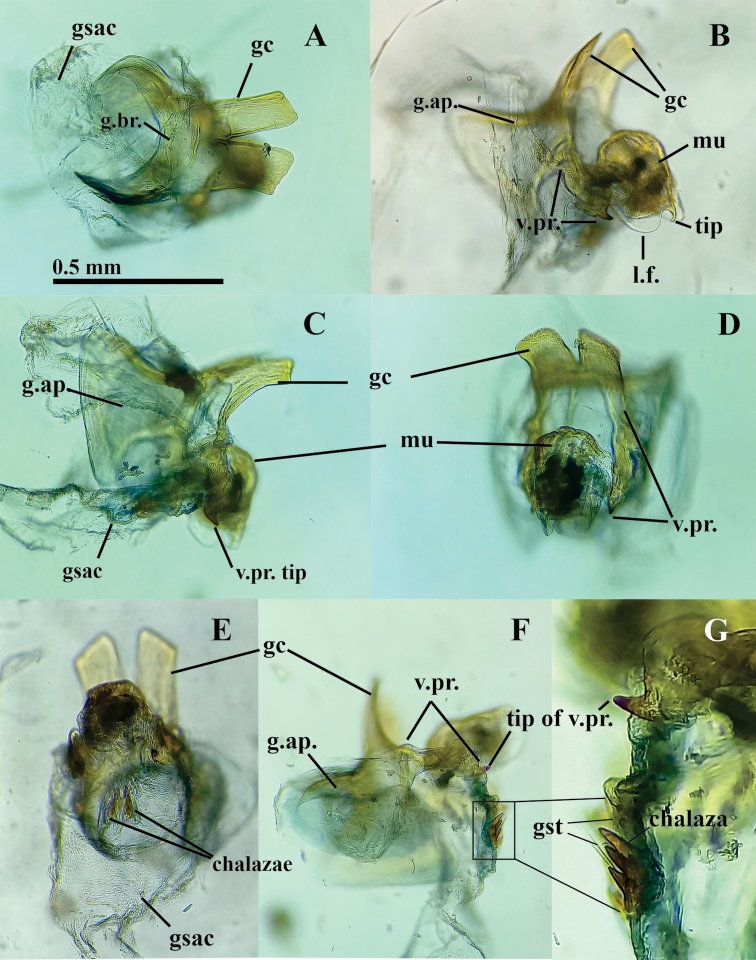
*Nuvolumbrosus* male gonarcal complex **A** dorsal **B** lateral slightly tilted to left **C** lateral slightly tilted to right **D, E** frontal **F** lateral (field of chalazate setae in box] **G** enlarged frontal section of gonosaccus, lateral. **Note: F, G** illustrate the placement and structure of the acute tip of the gonarcal ventral projection and the frontal section of the gonosaccus bearing a group of three heavily sclerotized chalazae with fine setae. Abbreviations: **g.ap.** gonarcal apodeme; **g.br.** gonarcal bridge; **gc** gonocornu; **gsac** gonosaccus; **gst** gonosetae; **l.f.** lateral flank of mediuncus; **mu** mediuncus; **tip of v.pr.** beaklike apex of gonarcal ventral projection; **v.pr.** ventral projection of gonarcus. Scale bar applies to **A–F**.

Note: The hypandrium internum can often be difficult to find. One was not found in this specimen. Either the specimen did not have one, it was not well developed, or it was lost.

***Abdomen, male*** (Figs 4–6): Yellowish with dark brown to black spots on tergites, sternites as follows: posterior sections of T1–T3, T6–T7, lateral margins of T1–T7, along dorsal apodeme below T8, tip of T9+ect, dorsal margin of S9 (Fig. 4A–C). Tergites, sternites quadrate, with all margins relatively straight, with long, robust setae, scattered short setae, dense microsetae, except S9 without microsetae (Fig. 5E); pleuron with sparse short setae except P7, P8 with setae large, dense. Microtrichiae covering the pleuron throughout. Spiracles small, round externally, atria not enlarged; rim sclerotized weakly. Callus cerci brown to black, round to slightly oval, located medially on T9+ect, with ~32 densely spaced trichobothria (Figs 4C, 5D). T9+ect fused dorsally, elongate, extending basally beneath T8 to distal margin of T7, with dorsal apodeme extending along full length of ventral margin, articulating basally with proximal end of ventral apodeme on dorsal margin of S8+9. Dorsal apodeme (Fig. 5A) strongly sclerotized throughout, bifurcated mesally, proximal to callus cerci, with dorsal spur almost reaching the dorsal margin of T9+ect, with lower section extending distally into setose lobe, well beyond distal margin of T9+ect (Fig. 5A–C). Basal section of S8+9 connected to T9+ect via membrane with scattered, long setae (Figs 4B, C, 5A). Dorsal margin of S8+9 (lateral view) with deep mesal cleft (Figs 4C, 5A); ventral surface of S8+9 with small suture-like separation, S8 with microsetae, S9 without microsetae (Fig. 5A). Dorsal margin of S8 with distinct apodeme (ventral apodeme) descending abruptly to base of cleft, covered by dense field of robust setae (Fig. 5A). Dorsal margin of S9 heavily convex, with sclerotized apodeme along upper edge, with round, sclerotized tips extending beyond end of segment (Fig. 5A–C, E). Sternites with ratio of maximum height / maximum length (lateral view): S2 = 0.5:1; S3 = 0.6:1; S4 = 0.8:1; S5 = 1:1; S6 = 1:1; S7 = 1.1:1; S8 = 1.5:1; S9 = 0.6:1; surfaces without microtholi.

Gonarcus well sclerotized, widely arcuate (maximum span 0.31 mm; minimum span between posterior apices of the lateral apodemes 0.28 mm); gonarcal bridge broad, curved, bearing two long, flat, quadrate gonocornua dorsally (~0.28 mm long, 0.14 mm wide), pair of broad oval-shaped gonarcal apodemes basally (0.46 mm tall, 0.22 wide); gonarcal bridge strongly fused with base of gonocornua (Fig. 6A), pair of ventral projections (~0.24 mm long) extending from the ventral surface of the gonarcal bridge (Fig. 6B–D, F), with distal area swollen, terminating in beak-like apex (Fig. 6B, C). Mediuncus attached to gonarcal bridge and ventral processes via membranes extending from lower surface of gonarcal bridge, from inner margins of ventral processes; dorsal surface of mediuncus apparently smooth (Fig. 6B–D), terminating distally in curved beak, flanked laterally by prominent lateral lobe (Fig. 6B). Gonosaccus with dorsal surface striate (Fig. 6E), with two mesal fields of three large, heavily sclerotized chalazae, each bearing one or two long, thin setae subapically (Fig. 6F, G); area on gonosaccus above heavy chalazae with additional smaller chalazate gonosetae (Fig. 6G). Hypandrium internum not seen.

***Abdomen, female***: Undescribed.

#### Immatures and biology.

Unknown.

#### Known geographic distribution.

Brazil: Rio de Janeiro, Minas Gerais (new record).

### ﻿ Nuvol satur

Taxon classificationAnimaliaNeuropteraChrysopidae

Sosa & Tauber sp. nov.

A74B76B3-B323-5C4B-9687-8CC1493D560E

https://zoobank.org/F4CCB95B-67EA-4A56-B5B5-5E89DE2FD422

#### Type specimens.

***Holotype***: Female, INPA; Brazil, Amazonia, Novo Aripuanã, 05°15'53"S, 60°07'08"W. Armadilha Malaise em igarapé; Floresta úmida, ix.2004, Henriques Silva & Pena leg. Specimen pinned. ***Paratype***: Female, EMUS; Brazil, Rondônia, 62 km SE Ariquemes, 7–18 Nov. 1995, W. J. Hanson.

#### Etymology.

The genus name “*Nuvol*” is a masculine noun meaning “cloud” in Catalan; the species name “*satur*” is a Latin adjective (masculine form) meaning “deep or full”, as applied to color (R. A. Pantaleoni, pers. comm.). The species name refers to the more intense coloration of the diffuse markings on the wings of the species, as compared with *N.umbrosus*.

#### Diagnosis.

**Figure 7.**
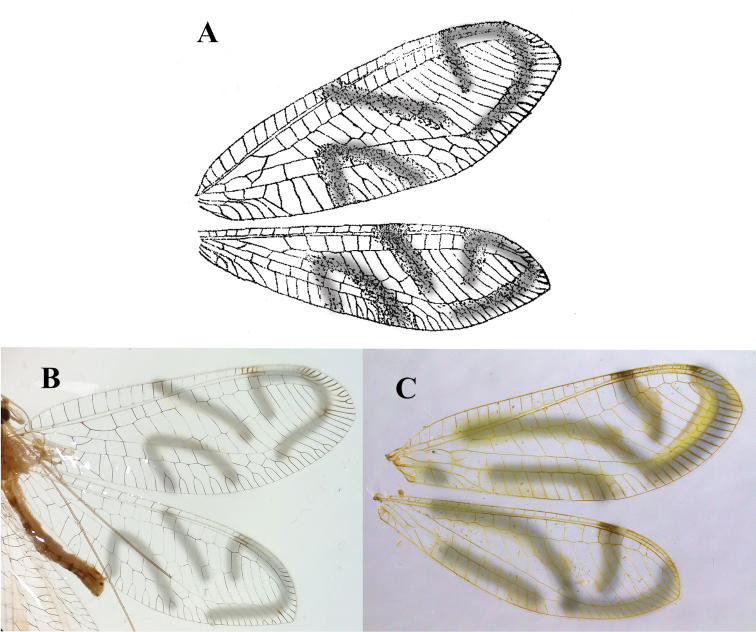
Cartoon showing pattern of wing markings of two *Nuvol* species **A***N.umbrosus* type specimen (Brazil, Rio de Janeiro), from original drawing by Navás (1916)**B***N.umbrosus* from current study (Brazil, Minas Gerais) **C***N.satur*, new species, holotype (Amazonas, Brazil), from Tauber and Sosa 2015 (as *N.umbrosus*).

The most notable features that distinguish *N.satur* from *N.umbrosus* are the head and pronotal markings, markings on the abdomen, wing size, and wing markings, as follows: (1) The head and prothoracic markings of *N.satur* are red and diffuse, whereas those of *N.umbrosus* are brown and longitudinally striped; (2) The wings of *N.satur* are 14.8–15.8 mm long, slightly shorter than those of *N.umbrosus* (17.0 mm); and (3) Although both *Nuvol* species express some degree of suppressed forking in the terminal veinlets of the forewings and hindwings, *N.satur* has a much greater degree of suppression than *N.umbrosus*. Almost none of the terminal veinlets of the *N.satur* wings are forked, whereas only a small proportion of the veinlets on the posterior margin of the *N.umbrosus* wings are unforked. Finally, (4) the wing markings of *N.satur* are considerably more pronounced and in a different pattern than those of *N.umbrosus* (Fig. 7).

#### Description.

Provided by Tauber and Sosa 2015: 144–150 (as *Nuvolumbrosus*). Note: In the description, we neglected to mention the length of the antennae; they measured 28.6 and 31.5 mm, over twice as long as the wing length (13.8 mm). The antennal length for *N.umbrosus* is unknown.

#### Immatures and biology.

Unknown.

#### Known geographic distribution.

Brazil: Amazonas, Rondônia.

### ﻿Genus Nuvol

Taxon classificationAnimaliaNeuropteraChrysopidae

Navás, 1916

C01826D3-97AA-5D3C-AB64-57DA21783E8A

#### Type species.

*Nuvolumbrosus* Navás, 1916.

#### Known geographic distribution.

South America: Brazil (Amazonas, Rondônia, Minas Gerais, Rio de Janeiro).

#### Generic diagnosis.

Based on a small number of specimens from two species:

*N.umbrosus* – one specimen of unknown sex described by Navás (1916) and one male described here.

*N.satur* – two females described by Tauber and Sosa (2015, as *N.umbrosus*).

Medium to large lacewings, forewing length 14.8–17.0 mm. Head, pronotum with longitudinal black stripes or diffuse reddish marks; setae long. Legs unmarked; claws basally dilated. Forewing marked with faint to dark yellowish-brown transverse streaks through center and margins of wing; costal area narrow throughout; costal setae short, inclined; stigma marked with one to two small dark spots; Sc and R well separated throughout; R extended apically, curving posteriorly around wing apex; terminal subcostal and radial veinlets at apex of wing largely unforked, darkly marked; *im* short, broadly ovate; Rs almost straight, parallel to R; radial cells short, height relatively uniform from base to below stigma; gradate veins arranged in two roughly parallel series; outer gradates closely aligned, flowing smoothly from PsM; inner gradates extending basally, not meeting PsM; four intracubital cells, with *icu1*, *icu2*, *icu3* closed, *icu4* (*dcc*) open. Hindwing venation, markings similar to forewing.

**Possible additional generic features, with supporting evidence from only one species and/or one specimen**: Antennae very long (over twice length of forewing). ***Female***: T9+ect separated dorsally by longitudinal groove. Spermatheca doughnut shaped, with elongate narrow spermathecal duct, substantial, sail-like velum opening directly to bursa copulatrix via dorsal slit. Bursa copulatrix with delicate membrane, elongate bursal glands. Subgenitale substantial, with bilobed knob protruding from broad triangular base. ***Male***: T9+ect with prominent, heavily sclerotized, bifurcated dorsal apodeme: with dorsal spur extending upward behind and well above callus cerci, with ventral branch extending distally, protruding as lobe well beyond distal margin of ectoproct. T9+ect fused dorsally; callus cerci round to very slightly oval, dark against pale background. Sternites S8, S9 weakly fused, with conspicuous cleft or suture scars. Gonarcus well sclerotized, widely arcuate; bridge broad, curved, with pair of elongate ventral projections extending ventrally; gonocornua long, broad. Mediuncus bulbous basally, with slender terminus, membranous dorsal attachment to gonarcal bridge, lateral attachments to inner sides of ventral projections of gonarcus.

## ﻿Generic relationships

The largely Neotropical green lacewing tribe Leucochrysini currently contains ~190 species classified into seven genera. One very large genus (*Leucochrysa*), with its two subgenera, accounts for the vast majority of leucochrysine species. Other species are distributed among a midsized genus of eight described species and five genera with only one or two species each (Brooks and Barnard 1990; Tauber et al. 2008; Oswald 2013). Although the tribe itself appears to be monophyletic, relationships within the group are largely unresolved (e.g., Garzón-Orduña et al. 2019; Winterton et al. 2019; Breitkreuz et al. 2021). In its original description (Navás 1916) and in subsequent discussion (Brooks and Barnard 1990; Tauber and Sosa 2015), *Nuvol* was distinguished from *Leucochrysa* and other leucochrysine genera largely on the basis of forewing features, notably: an elongate radius that parallels the subcosta as it extends along the length of the wing and curves upward at the tip of the wing; terminal veins at the apex of the wing largely unforked; outer gradates aligned with neighboring gradates in a smooth trajectory that parallels the wing margin; an elongate, marked stigma; and four intracubital cells, rather than the typical three. Most noticeable are the distinctively diffuse and patterned markings on the forewings and hindwings. Both *Nuvol* species now known express this full suite of character states, but most of the features do not appear to be unique to the genus. For example, although most Leucochrysines that have been studied have three intracubital cells, the pattern of four intracubital cells that typifies *Nuvol* is also present in *Berchmansus* spp. [now assigned to Leucochrysini (Tauber 2007)] and in *Nothancylaverreauxi* Navás [previously assigned to Leucochrysini by Brooks & Barnard (1990), now tentatively assigned to Apochrysini by Winterton & Brooks (2002)]. Similarly, the linear alignment of the outer gradates and their flow into the PsM can be seen in most Leucochrysa (L.) species [notable examples: L. (L.) boxi Navás, L. (L.) nigrilabris (Banks), L. (L.) insularis (Walker) (Tauber et al. 2011a, b; Tauber et al. 2013)]. However, although diffused markings and streaks on the forewings are also found in other leucochrysine genera such as *Gonzaga*, Leucochrysa (Nodita), and *Santocellus* (see Brooks and Barnard 1990; Tauber et al. 2008, 2011b; Tauber 2012), they are usually not found on the hindwings and their patterns differ from those of the *Nuvol* species. And finally, unforked terminal veins at the apex of the forewing are unusual among Leucochrysini. So, at this time, we retain *Nuvol* as a distinct genus, while simultaneously acknowledging that the intriguing characters, and the frustrating lack of information associated with leucochrysine lacewings in general, provide stimulus for future investigation.

## Supplementary Material

XML Treatment for Nuvol umbrosus

XML Treatment for Nuvol satur

XML Treatment for Nuvol
